# HAMSIAA protects cyclophosphamide induced immunosuppression by modulating gut homeostasis and purine metabolism

**DOI:** 10.3389/fimmu.2026.1925823

**Published:** 2026-09-03

**Authors:** Di Zhang, Yingying Song, Shihao Guo, Xiaoqian Cui, Hongjing Dong, Qingjun Li, Quanbo Wang, Charles R. Mackay, Xinyan Qu

**Affiliations:** 1 Key Laboratory for Natural Active Pharmaceutical Constituents Research in Universities of Shandong Province, School of Pharmaceutical Sciences, Shandong Analysis and Test Center, Qilu University of Technology (Shandong Academy of Sciences), Jinan, China; 2 Shandong Shanwei Immunotech Co., Ltd., Jinan, China; 3 Shandong University of Traditional Chinese Medicine, Jinan, China

**Keywords:** CTX, gut microbiota, HAMSIAA, immunomodulation, immunosuppression, purine metabolism

## Abstract

**Introduction:**

Dietary supplementation with functional starches serves as a promising intervention strategy for immune regulation, yet the immunomodulatory efficacy and underlying mechanisms of newly developed modified starch derivatives remain to be further explored. In this study, a cyclophosphamide (CTX) induced secondary immunodeficiency mouse model was adopted to investigate the immunoregulatory effects of indole 3 acetylated high amylose maize starch derivative (HAMSIAA).

**Methods:**

Mice were fed a basal diet supplemented with 0.5% or 1% HAMSIAA for nine consecutive days, with CTX administered starting on day 7. Immune organ injury, splenic CD4⁺/CD8⁺ T cell balance, serum immunoglobulins, intestinal barrier integrity, gut microbiota composition and purine related metabolome were assessed.

**Results:**

HAMSIAA attenuated CTX triggered damage to immune organs, maintained the splenic CD4⁺/CD8⁺ T cell balance, and increased serum levels of Immunoglobulin A (IgA), immunoglobulin G (IgG), and immunoglobulin M (IgM). In addition, HAMSIAA improved intestinal barrier integrity and modulated gut microbiota composition, as evidenced by changes in the Bacillota/Bacteroidota (B/B) ratio and a reduction in Pseudomonadota abundance. Metabolomic analyses further revealed that HAMSIAA was associated with modulation of the purine salvage pathway, including downregulation of hypoxanthine guanine phosphoribosyltransferase (HGPRT) and altered levels of metabolites such as inosine and xanthine, which may contribute to its immunoprotective effects.

**Discussion:**

Collectively, the present findings suggest that HAMSIAA is a promising functional food ingredient for the prophylactic mitigation of chemotherapy induced immunosuppression, and provide support for its translational application in the field of immunonutrition.

## 1 Introduction

The immune system plays an essential role in defending the host from infections and sustaining bodily homeostasis (1). Immunosuppression leads to decreased pathogen resistance and disrupted immune homeostasis, increasing the risk of infections, autoimmune diseases, and tumorigenesis. CTX, a common chemotherapeutic drug, is widely utilized to construct mouse models of chemotherapy-induced secondary immunodeficiency, which recapitulate the immunocompromised state observed in clinical chemotherapy patients (2, 3). Such an animal model offers a reliable platform to explore the immunomodulatory capacity of HAMSIAA and dissect its underlying molecular mechanisms.

IAA is a classic plant auxin and a tryptophan-derived metabolite generated by intestinal commensal bacteria including *Bacteroides* and *Clostridium* spp., which acts as a pivotal ligand for the aryl hydrocarbon receptor (AhR). IAA regulates the differentiation and activation of host immune cells: it drives CD4^+^ T cells to reprogram into intestinal CD4^+^CD8αα^+^ regulatory T cells, maintaining intestinal antigen tolerance (4). In addition, IAA cooperates with lipopolysaccharide to induce IL-35^+^ regulatory B cells via the TLR4–NFκB cascade, further facilitating regulatory T cell proliferation and suppressing the differentiation of proinflammatory Th1/Th17 subsets (5). Furthermore, IAA activates AhR signaling and upregulates IL-22, ZO-1, and Claudin-1 expression in tissue, protecting against colitis and maintaining intestinal epithelial homeostasis (6). Collectively, IAA exerts a vital function in sustaining intestinal immune homeostasis and restraining excessive inflammatory responses (7, 8).

Previous studies have shown that wild ginseng restores tryptophan metabolites including IAA and alleviates CTX-induced immunosuppression (9). However, the therapeutic potential of standalone IAA against immunodeficiency and its detailed molecular basis are poorly characterized. Our previous work successfully synthesized HAMSIAA and verified its capacity to elevate endogenous IAA levels and its colon-targeted delivery in mice (10). However, whether HAMSIAA exerts direct immunomodulatory effects beyond colonic protection, especially under systemic immunosuppressive conditions, remains unexplored. Notably, untargeted metabolomics analysis in this study revealed that purine metabolism, particularly inosine levels, was significantly altered by HAMSIAA. Given that inosine is known to regulate immune homeostasis and maintain intestinal barrier integrity (11, 12), this observation implies a potential functional crosstalk between HAMSIAA-upregulated IAA and purine metabolic pathways. Herein, we systematically investigated the prophylactic protective effects of HAMSIAA against CTX-induced immunosuppression together with its intrinsic molecular mechanisms. Collectively, this work provides a mechanistic basis for the immunoprotective effects of HAMSIAA and supports its further development as a functional food ingredient for the prophylactic mitigation of chemotherapy-associated immunosuppression.

## 2 Materials and methods

### 2.1 Materials and reagents

CTX (batch no. DKC574) was purchased from Shanghai Bide Pharmaceutical Technology Co., Ltd. HAMSIAA, a modified starch derivative based on high-amylose maize starch, was prepared in our laboratory as previously described (10). Enzyme-linked immunosorbent assay (ELISA) kits for mouse IgG, IgM, and IgA (batch nos. 274466-012, 271117-015, and 271791-007) were purchased from Thermo Fisher Scientific Inc. Antibodies against CD3, CD4, and B220 were purchased from Invitrogen, and anti-CD8 antibody was purchased from BD Biosciences (USA).

### 2.2 Animals and treatments

All animal experimental procedures were approved by the Experimental Animal Welfare and Ethics Committee of Shandong University of Traditional Chinese Medicine (approval No. SDUTCM20260228501). Male C57BL/6J mice (7–8 weeks old) were purchased from Shandong Pengyue Experimental Animal Technology Co., Ltd. (Shandong, China). Mice were housed under controlled environmental conditions (22 ± 2 °C, 12 h light/dark cycle) with free access to food and water. After acclimatization, mice were randomly divided into five groups (n = 8 per group): normal control (NC), CTX model, levamisole hydrochloride (LH) positive control, 0.5% HAMSIAA, and 1% HAMSIAA groups. Mice were fed a basal diet supplemented with 0.5% or 1% HAMSIAA from day 1 to day 9. Following 6 days of HAMSIAA pre-feeding, mice in all groups except the NC group received intraperitoneal injections of CTX (80 mg/kg) on days 7–9. Mice in the LH group were orally administered LH (60 mg/kg) on days 7–9, while those in the NC group received an equal volume of sterile saline by gavage. Body weights were recorded 12 h after the last administration on day 9. Anesthesia was induced by intraperitoneal injection of a mixture of tribromoethanol (200 mg/kg) and xylazine (20 mg/kg). Once deep anesthesia was confirmed, blood samples were collected via enucleation of the eyeballs, and mice were subsequently euthanized by cervical dislocation. Spleen, thymus, and ileal tissues were harvested for subsequent analyses.

### 2.3 Determination of spleen and thymus indices

Spleen and thymus were harvested and weighed immediately after dissection. Organ indices were calculated as: Organ index (mg/g) = Organ weight (mg)/Body weight (g) × 100.

### 2.4 Histology examination

Spleen and ileum tissues were fixed in 4% neutral paraformaldehyde for 24 h, dehydrated through a graded ethanol series, cleared in xylene, and embedded in paraffin. Tissue blocks were sectioned at 5 μm thickness. Sections were deparaffinized, rehydrated, stained with hematoxylin and eosin (H&E), and mounted with neutral balsam. Histomorphological changes were observed under a light microscope. Blinded histological scoring was performed based on four parameters, including inflammatory cell infiltration, villus integrity, crypt morphology, and mucosal edema. Each parameter was scored on a scale of 0-4, giving a total score range of 0-16), with higher scores reflecting more severe ileal injury.

### 2.5 Immunohistochemistry staining of ileal tissues

Paraffin-embedded ileal tissue sections were routinely dewaxed, followed by antigen retrieval, endogenous peroxidase blocking, and blocking with blocking buffer. The sections were then incubated with primary antibodies against Occludin (1:250) and ZO-1 (1:1000) overnight at 4 °C, followed by incubation with secondary antibodies and DAB chromogen development on the next day. Nuclei were counterstained with hematoxylin, and the sections were dehydrated, mounted with neutral balsam, and allowed to dry before observation and photography under a microscope. Semi-quantitative analysis of ZO-1 and Occludin positively stained areas was performed using ImageJ software in a blinded manner.

### 2.6 Preparation of splenocytes and flow cytometry analysis

Mouse spleens were isolated and ground to prepare single-cell suspensions. After red blood cell lysis and centrifugation, cells were stained with antibodies against CD3, CD4, CD8, B220 and 7-AAD in the dark at 4 °C for 40 min. After washing, flow cytometric analysis was performed, and data were processed using FlowJo V10 software.

### 2.7 Determination of serum immunoglobulins

Serum immunoglobulin levels were quantified using ELISA in strict accordance with the manufacturer’s instructions. Briefly, serum samples were incubated at 4 °C for complete clotting, followed by centrifugation at 12,000×g for 10 min at 4 °C. Serum samples were collected to quantitatively determine the concentrations of IgG, IgM, and IgA in different experimental groups.

### 2.8 Quantitative real-time PCR analysis

Total RNA was extracted by Trizol method, followed by chloroform extraction, isopropanol precipitation, and 75% ethanol washing, then dissolved in RNase-free water. RNA purity was determined using a UV spectrophotometer. cDNA was synthesized from total RNA using the SPARKscript II RT Plus kit. qRT-PCR was performed on a Roche LightCycler 480 system with SYBR Green, using GAPDH as the internal control. The relative expression of *IL-1β*, *TNF-α*, *APRT*, *HGPRT*, *ZO-1*, *Occludin*, and *CYP1A1* was calculated by the 2^-^ΔΔCt method. *APRT* and *HGPRT* mRNA levels were measured in thymus tissues, *IL-1β*, *TNF-α*, *ZO-1*, *Occludin*, *and CYP1A1* mRNA were measured in ileal tissues.

### 2.9 Gut microbiota analysis

Fecal microbial DNA was extracted from fecal samples. 16S rRNA sequencing was performed on fecal samples from the NC, CTX, and 1% HAMSIAA groups (n = 6 per group). The V3–V4 hypervariable regions of the 16S rRNA gene were amplified, and PCR products were purified and sequenced on the Illumina platform. Raw reads were subjected to quality filtering, assembly, and denoising to generate amplicon sequence variants (ASVs), followed by taxonomic annotation. Alpha diversity was assessed using Chao1 and Shannon indices, and beta diversity was evaluated via non-metric multidimensional scaling (NMDS) and principal component analysis (PCA). The relative abundance of gut microbiota and the Bacillota/Bacteroidota(B/B) ratio were calculated and statistically compared. LEfSe analysis was performed to identify differentially abundant taxa between groups, with an LDA score threshold > 2 and p < 0.05 considered statistically significant.

### 2.10 Untargeted metabolomics analysis of serum by UPLC-MS/MS

Serum metabolites were extracted by gradual thawing of samples, followed by addition of prechilled methanol/acetonitrile (1:1, v/v). Untargeted metabolomics analysis was performed on serum samples from the NC, CTX, and 1% HAMSIAA groups (n = 8 per group). After ultrasonication and protein precipitation, samples were centrifuged and supernatants were dried under nitrogen gas and reconstituted. The reconstituted solution was centrifuged and filtered prior to LC-MS/MS analysis. Quality control samples were prepared by pooling equal volumes of each sample to monitor analytical stability.

### 2.11 Statistical methods

All statistical analyses were conducted using GraphPad Prism 8.2.1. Two-group comparisons were performed using unpaired two-tailed Student’s t-test to determine statistical significance. All graphs are presented as mean ± standard error of the mean (SEM). For correlation analyses, Pearson’s correlation coefficient was calculated. Statistical significance was defined as *p* < 0.05. In the figures, #*p* < 0.05, ##*p* < 0.01, and ###*p* < 0.001 indicate comparisons versus the NC group; *p < 0.05, **p < 0.01, and ***p < 0.001 indicate comparisons versus the CTX group. All significance markers are defined in the corresponding figure legends.

## 3 Results

### 3.1 HAMSIAA attenuated CTX-induced weight loss and improved splenic and thymic indices in mice

**Figure 1:**
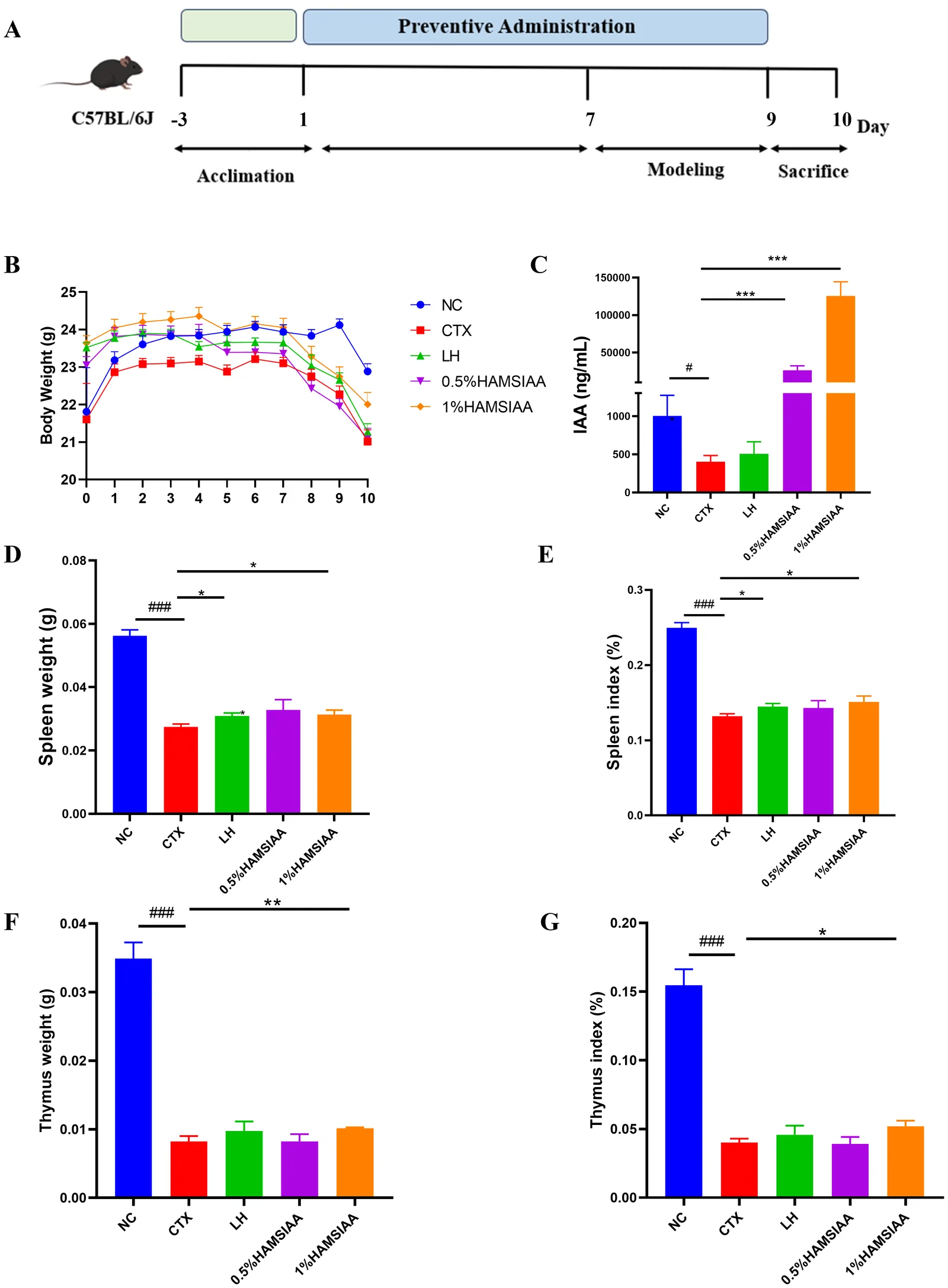
Effects of HAMSIAA on body weight, thymic index, and splenic index in immunocompromised mice. **(A)** Schematic diagram of mouse feeding and experimental procedure. **(B)** Changes in body weight of mice. **(C)** IAA content in mouse feces. **(D)** Spleen weight of mice. **(E)** Splenic index of mice. **(F)** Thymus weight of mice. **(G)** Thymic index of mice. Data are presented as mean ± SEM (n = 8 per group). ^#^*p* < 0.05, ^##^*p* < 0.01, ^###^*p* < 0.001 versus NC group; *p < 0.05, **p < 0.01, ***p < 0.001 versus CTX group.

In this study, an immunosuppressed murine model was established by intraperitoneal injection of CTX for three consecutive days. Mice were fed a basal diet supplemented with 0.5% or 1% HAMSIAA on days 1–9, and received CTX injections on days 7–9 to induce an immunosuppressive model (**Figure 1A**). CTX-induced immunosuppressed mice exhibited a significant reduction in fecal IAA compared with normal controls (*p* < 0.05). Both 0.5% and 1% HAMSIAA supplementation effectively elevated fecal IAA levels (*p* < 0.001, **Figure 1B**).

Body weight, spleen index and thymus index are validated surrogate markers of immunocompetence in CTX-induced immunosuppression (13). Compared with the NC group, CTX-treated mice exhibited significant weight loss and marked decreases in spleen and thymus weights and their respective indices (*p* < 0.001). However, 1% HAMSIAA effectively attenuated CTX-induced weight loss. Both 0.5% and 1% HAMSIAA significantly increased spleen weight and spleen index (*p* < 0.05), whereas 1% HAMSIAA additionally attenuated the decline in thymus weight and thymus index (*p* < 0.05, **Figures 1C–G**). Collectively, HAMSIAA attenuated CTX-induced atrophy of primary and secondary immune organs.

### 3.2 Effects of HAMSIAA on splenic histopathology and immunoglobulin levels in immunosuppressed mice

**Figure 2:**
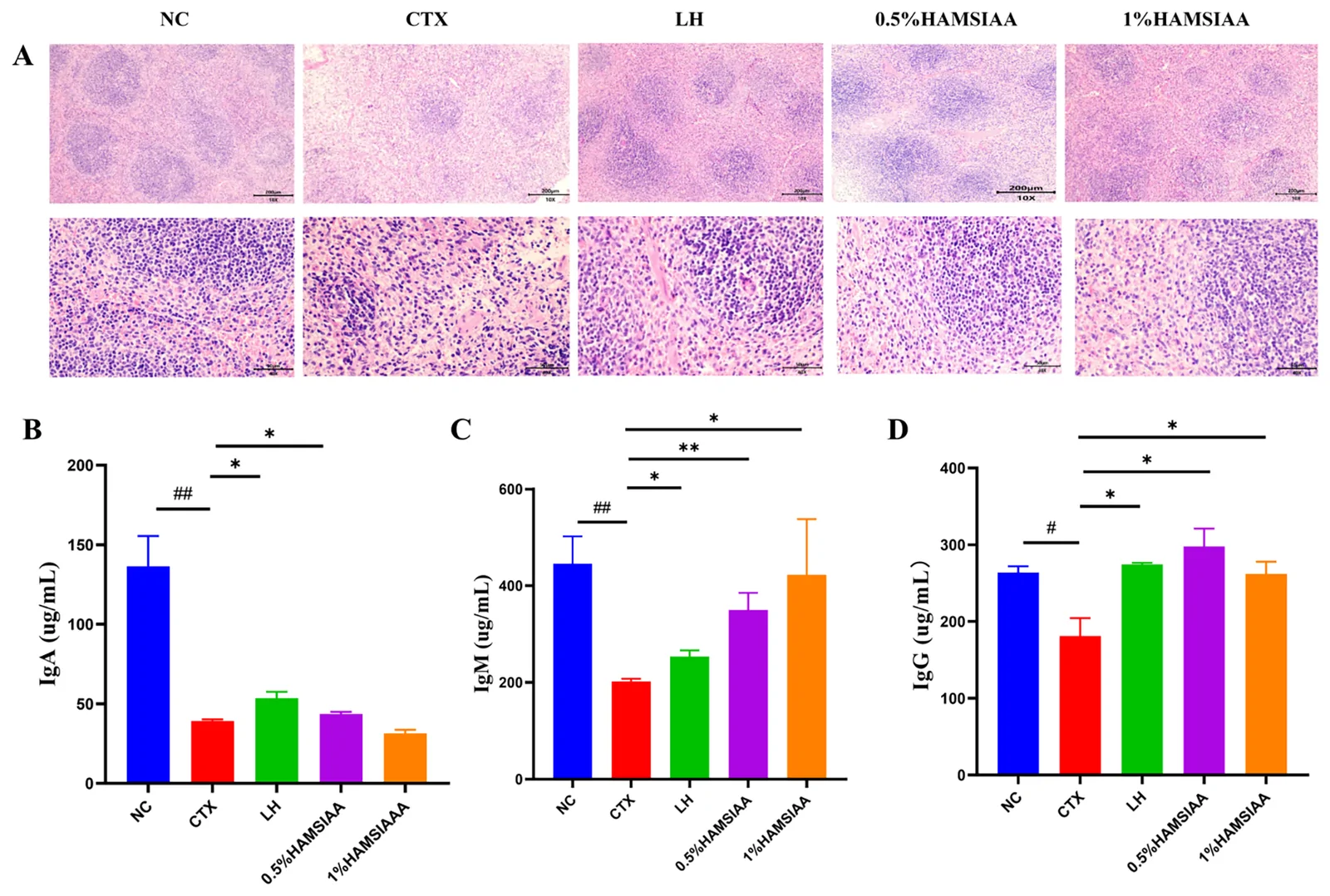
HAMSIAA attenuates CTX-induced splenic pathological damage in immunodeficient mice. **(A)** Representative H&E-stained images of splenic tissues. Upper panel (scale bar: 200 μm); lower panel (scale bar: 50 μm). **(B)** Serum IgA levels. **(C)** Serum IgM levels. **(D)** Serum IgG levels. Data are presented as mean ± SEM (n = 8 per group). ^#^*p* < 0.05, ^##^*p* < 0.01, ^###^*p* < 0.001 versus NC group; *p < 0.05, **p < 0.01, ***p < 0.001 versus CTX group.

To further evaluate the modulatory effects of HAMSIAA on immune organs and humoral immunity in immunosuppressed mice, splenic histoarchitecture and serum immunoglobulin (IgA, IgG, and IgM) levels were assessed. Histological examination of H&E-stained spleen sections revealed that NC mice exhibited a well-defined splenic architecture with distinct white and red pulp, prominent lymphoid follicles, and a clearly demarcated marginal zone. In contrast, CTX-treated mice displayed disruption of the white–red pulp boundary and disorganization of splenic follicles. Dietary supplementation with 0.5% or 1% HAMSIAA substantially attenuated these pathological alterations (**Figure 2A**). Compared with the NC group, CTX challenge significantly reduced serum IgA, IgG, and IgM levels (*p* < 0.05), while HAMSIAA supplementation dose-dependently elevated immunoglobulin levels (**Figures 2B–D**) (*p* < 0.05).

Collectively, these data demonstrate that HAMSIAA supplementation attenuates CTX-mediated splenic pathology and elevates serum immunoglobulin levels.

### 3.3 Effects of HAMSIAA on splenic immune cells

**Figure 3:**
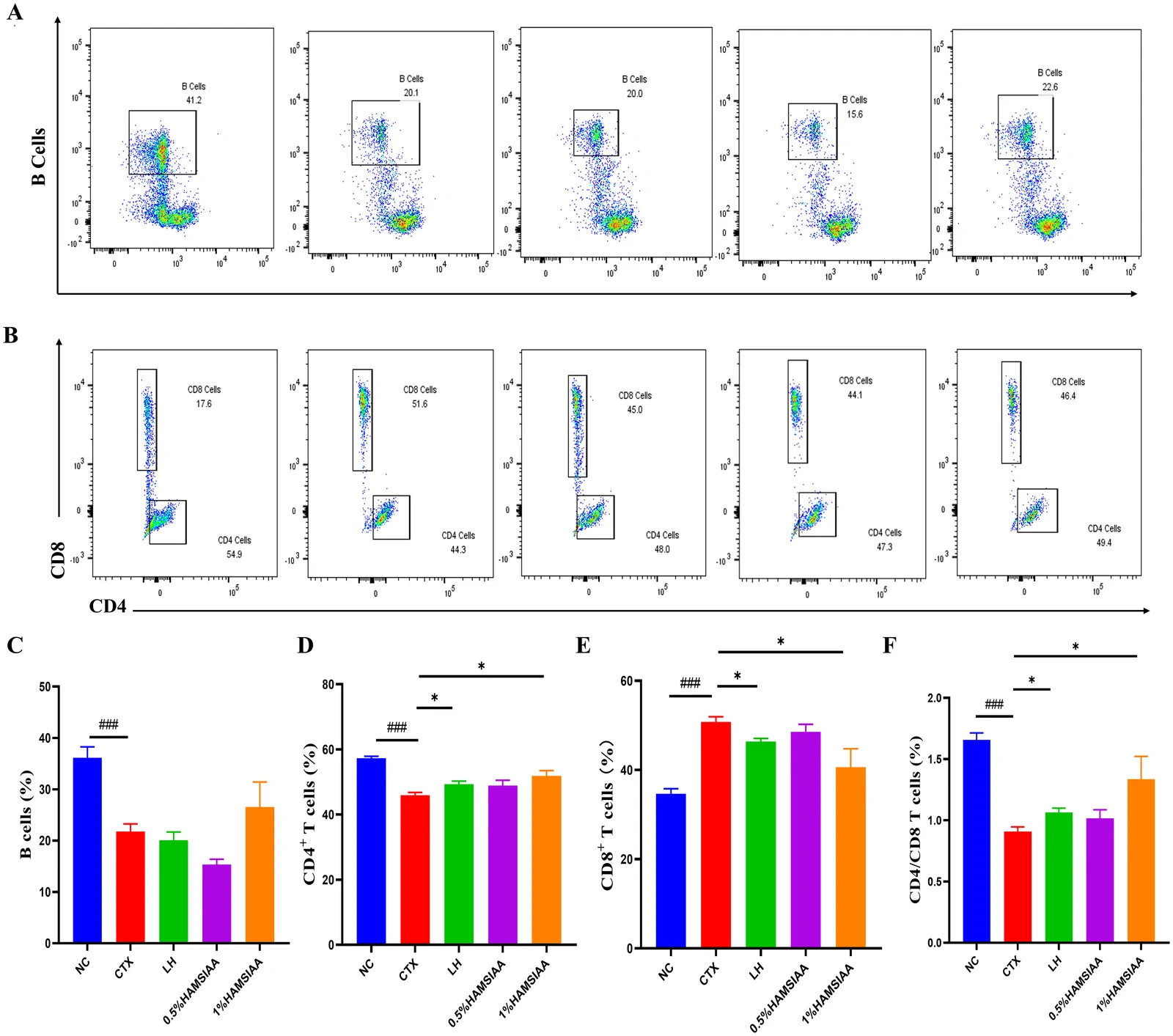
Effect of HAMSIAA on splenic immune cells populations in immunodeficient mice. **(A, B)** Representative flow cytometry plots of splenic B cells, CD4^+^ T cells, and CD8^+^ T cells. **(C–E)** Effects of HAMSIAA on the proportions of B cells, CD4^+^ T cells, and CD8^+^ T cells in mouse spleen; effect of HAMSIAA on the CD4^+^/CD8^+^ T cell ratio in mouse spleen. Data are presented as mean ± SEM (n = 6 per group). ^#^*p* < 0.05, ^##^*p* < 0.01, ^###^*p* < 0.001 versus NC group; *p < 0.05, **p < 0.01, ***p < 0.001 versus CTX group.

Compared with the NC group, CTX treatment significantly decreased the percentage of B cells (*p* < 0.01) and CD4^+^ T cells (*p* < 0.01), while significantly increasing the percentage of CD8^+^ T cells (*p* < 0.01), with a consequent reduction in the CD4^+^/CD8^+^ ratio (*p* < 0.01). Compared with the CTX group, intervention with 1% HAMSIAA led to an increase in the percentage of B cells, although this difference did not reach statistical significance. In contrast, 1% HAMSIAA significantly increased the percentage of CD4^+^ T cells, significantly decreased the percentage of CD8^+^ T cells, and significantly attenuated the reduction in the CD4^+^/CD8^+^ ratio (*p* < 0.05) (**Figure 3**).

### 3.4 Protective effects of HAMSIAA against CTX-induced ileal injury

**Figure 4:**
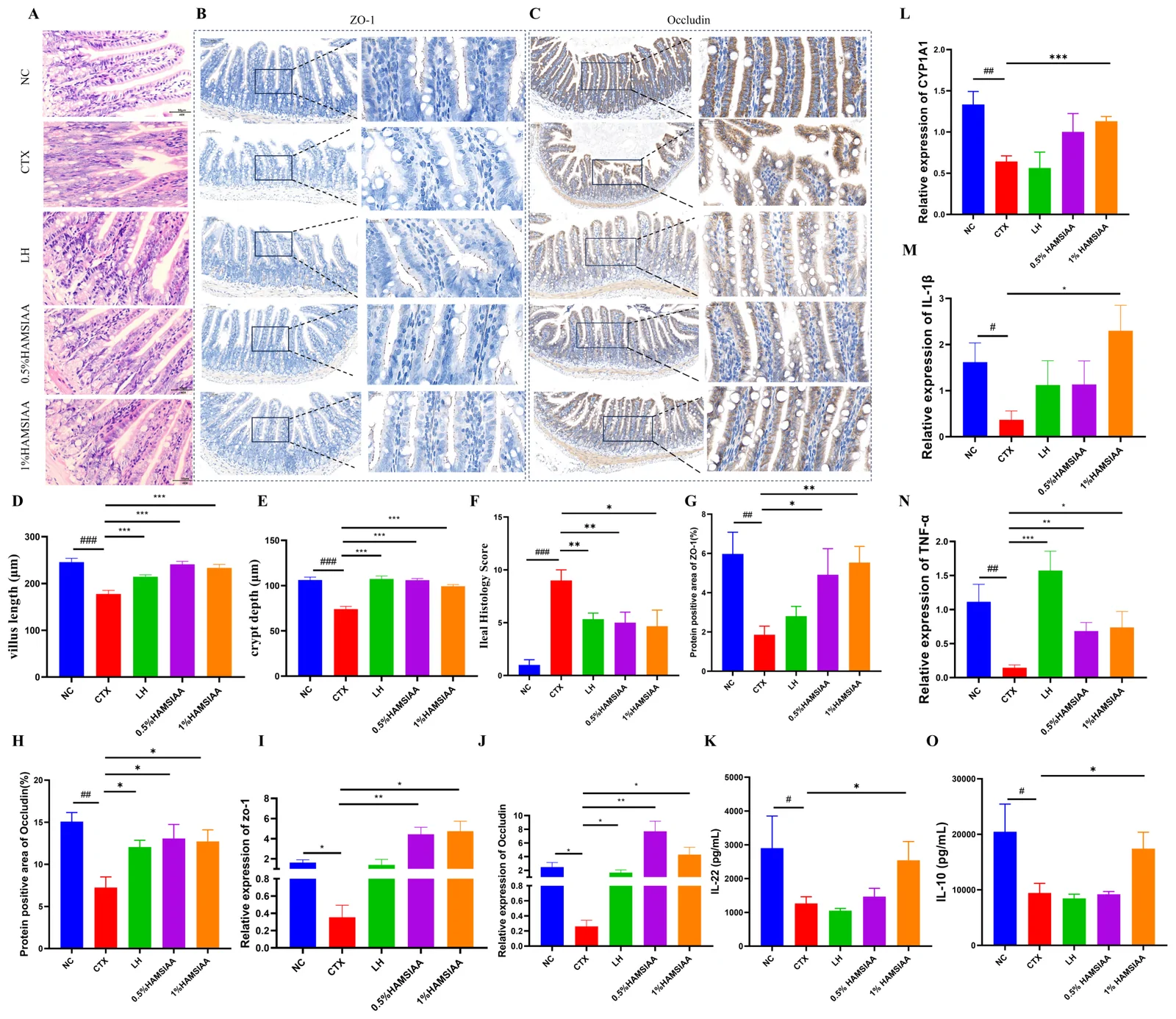
Protective effects of HAMSIAA against CTX-induced ileal injury in immunodeficient mice. **(A)** Representative H&E-stained images of mouse ileal tissues, scale bar = 50 μm. **(B)** Representative immunohistochemical staining images of ZO-1 in ileal tissues, panoramic low-magnification view scale bar = 100 μm, magnified inset scale bar = 20 μm. **(C)** Representative immunohistochemical staining images of Occludin in ileal tissues, panoramic low-magnification view scale bar = 100 μm, magnified inset scale bar = 20 μm. **(D)** Quantitative analysis of ileal villus length. **(E)** Quantitative analysis of ileal crypt depth. **(F)** Histological inflammatory pathology score of ileal tissues. **(G)** Quantitative analysis of ZO-1 positive area percentage. **(H)** Quantitative analysis of Occludin positive area percentage. **(I)** *ZO-1* mRNA expression level in ileal tissues. **(J)** *Occludin* mRNA expression level in ileal tissues. **(K)** IL-22 protein levels in ileal tissues measured by ELISA. **(L)** *CYP1A1* mRNA expression level in ileal tissues. **(M)** *IL-1β* mRNA expression level in ileal tissues. **(N)** *TNF-α* mRNA expression level in ileal tissues. **(O)** IL-10 protein levels in ileal tissues measured by ELISA. Data are presented as mean ± SEM (qPCR and ELISA: n = 8 per group; histomorphology and immunohistochemistry: n = 3 per group). ^#^*p* < 0.05, ^##^*p* < 0.01, ^###^*p* < 0.001 versus NC group; *p < 0.05, **p < 0.01, ***p < 0.001 versus CTX group.

Histopathological evaluation of ileal tissue was performed using H&E staining (**Figure 4A**). In the NC group, the ileal architecture was intact with well-aligned and densely arranged crypts, whereas the CTX group exhibited pronounced pathological damage, characterized by inflammatory cell infiltration, significant reductions in villus length and crypt depth (**Figures 4D, E**), and a marked increase in histological inflammatory pathology score (**Figure 4F**) (*p* < 0.01). Dietary supplementation with 0.5% or 1% HAMSIAA significantly attenuated these CTX-induced histological lesions and protected against mucosal damage.

To verify the expression changes of tight junction proteins at both the transcriptional and tissue levels, qRT-PCR and immunohistochemical staining of ZO-1 and Occludin were performed (**Figures 4B, C**). qRT-PCR analysis revealed that CTX exposure significantly downregulated ileal transcript levels of *ZO-1* and *Occludin* relative to the NC group (*p* < 0.05), while dietary supplementation with either 0.5% or 1% HAMSIAA significantly preserved their mRNA expression (**Figures 4I, J**). Immunohistochemical staining further confirmed these findings: in the NC group, continuous and clear ZO-1 and Occludin positive staining (brown-yellow) was observed along the margins of intestinal epithelial cells, with intact distribution along the cell membrane. In contrast, the CTX group exhibited fragmented tight junction staining and extensive loss of positive signals, with the positively stained area significantly reduced compared with the NC group (**Figures 4G, H**). In the 0.5% and 1% HAMSIAA groups, the continuous staining structure of ZO-1 and Occludin was markedly preserved, and the positively stained area was significantly increased compared with the CTX group. In the LH group, ZO-1 staining trended to increase but did not reach statistical significance, while Occludin staining was significantly preserved.

We further examined the effect of HAMSIAA on AhR pathway activation in the ileum. CTX treatment significantly reduced ileal IL-22 protein levels and downregulated *CYP1A1* mRNA expression compared with the NC group (*p* < 0.05), while HAMSIAA supplementation significantly elevated IL-22 levels and upregulated *CYP1A1* expression (**Figures 4K, L**) (*p* < 0.05). Cytokine analysis further revealed that CTX challenge significantly reduced ileal IL-1β and TNF-α mRNA expression levels compared with the NC group (*p* < 0.05); in contrast, 0.5% HAMSIAA maintained *TNF-α* mRNA levels, while 1% HAMSIAA significantly preserved both *IL-1β* and *TNF-α* mRNA levels (**Figures 4M, N**) (*p* < 0.05). In addition, we measured IL-10 protein levels in ileal tissues by ELISA. CTX treatment significantly reduced ileal IL-10 levels compared with the NC group (*p* < 0.05), while 1% HAMSIAA supplementation significantly elevated IL-10 levels (*p* < 0.05), with a similar but non-significant trend observed in the 0.5% dose group (**Figure 4O**).

### 3.5 HAMSIAA modulated gut microbiota diversity and community structure in CTX-induced mice

**Figure 5:**
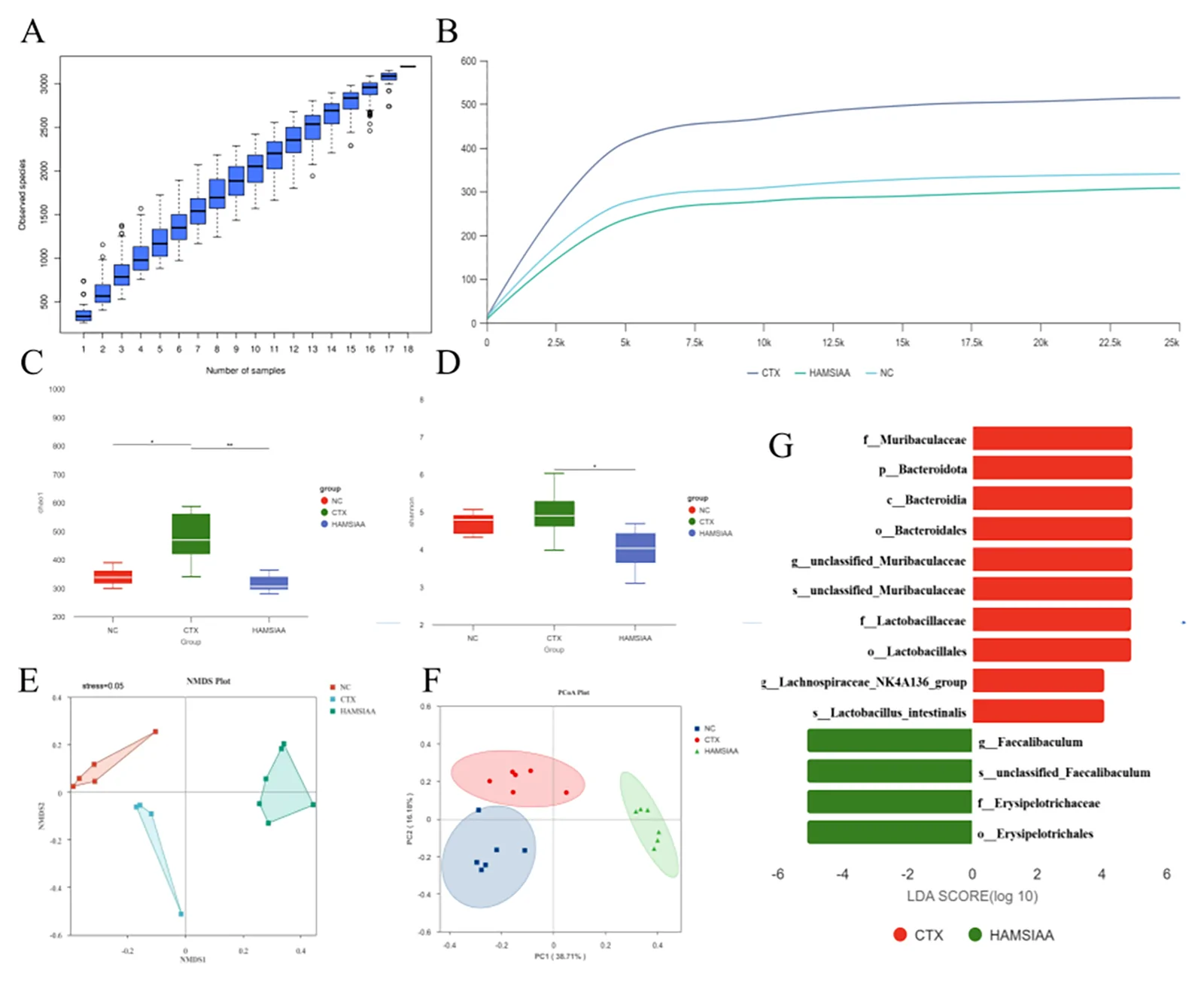
Analysis of gut microbiota diversity in mice. **(A)** Species accumulation curve. **(B)** Rarefaction curve. **(C)** α-Diversity analysis based on Chao1 index. **(D)** α-Diversity analysis based on Shannon index. **(E)** β-Diversity analysis based on NMDS. **(F)** β-Diversity analysis based on PCA. **(G)** LEfSe analysis revealing differentially abundant taxa between the CTX and 1% HAMSIAA groups (LDA score > 2, p < 0.05). For figures C and D, statistical significance was determined using the platform’s default algorithm. Data are presented as mean ± SEM (n = 6 per group).

A growing body of evidence has established a close association between compromised host immunity and intestinal dysbiosis (14). 16S rRNA sequencing was performed on fecal samples from the NC, CTX, and 1% HAMSIAA groups (n = 6 per group). Based on this premise, we employed 16S rRNA sequencing to systematically characterize the taxonomic diversity, richness, and compositional architecture of the gut microbiota across the three experimental groups of mice. Species accumulation and rarefaction curves were generated to assess α-diversity with increasing sequencing depth. Both curves reached a plateau, indicating that the sequencing depth achieved was saturated and that the species present were evenly distributed across the samples (**Figures 5A, B**). Microbial α-diversity was quantified using the Chao1 and Shannon indices (**Figures 5C, D**). Compared with the NC group, the CTX-treated group exhibited a significant increase in the Chao1 index (*p* < 0.01). However, HAMSIAA intervention significantly attenuated this elevation, resulting in the Chao1 index significantly lower than that of the CTX group (*p* < 0.01). β-diversity was evaluated by PCA and NMDS. Both ordinations revealed distinct, non-overlapping clusters corresponding to the three experimental groups, indicating a significant divergence in gut microbiota structure among the different treatments (**Figures 5E, F**).

To identify the specific taxa responsible for the CTX-induced increase in Chao1 richness, we performed LEfSe analysis. The elevated Chao1 index in CTX-treated mice was primarily associated with the significant enrichment of Bacteroidota and its representative family Muribaculaceae (LDA score > 2, *p* < 0.05) (**Figure 5G**). These findings are consistent with the decreased B/B ratio observed at the community level (**Figure 5E**), supporting the interpretation that CTX-induced immunosuppression permits the expansion of specific taxa, which may collectively contribute to the elevated Chao1 richness.

### 3.6 HAMSIAA attenuated the altered B/B ratio of gut microbiota in CTX-induced mice

**Figure 6:**
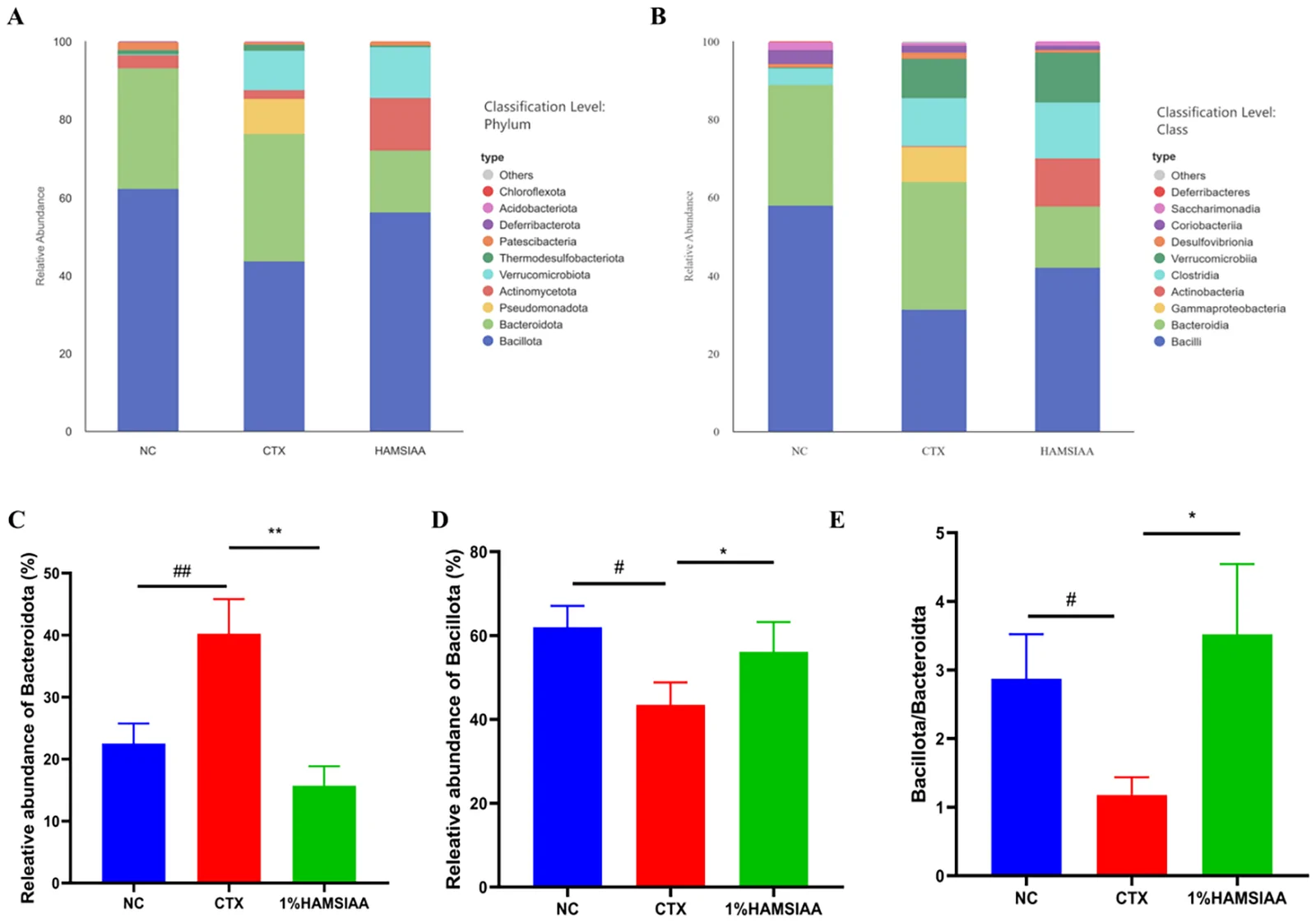
Analysis of gut microbiota composition and Bacillota/Bacteroidota (B/B) ratio in mice. **(A)** Relative abundance at the phylum level. **(B)** Relative abundance at the class level. **(C)** Relative abundance of Bacteroidota. **(D)** Relative abundance of Bacillota. **(E)** B/B ratio. Data are presented as mean ± SEM (n = 6 per group). ^#^*p* < 0.05, ^##^*p* < 0.01, ^###^*p* < 0.001 versus NC group; *p < 0.05, **p < 0.01, ***p < 0.001 versus CTX group.

Four dominant bacterial phyla were identified in murine fecal samples: Bacillota, Bacteroidota, Actinomycetota, and Verrucomicrobiota, with Bacillota and Bacteroidota accounting for the vast majority. Compared with the NC group, the CTX group exhibited a significant decrease in the relative abundance of Bacillota (*p* < 0.05) and a concomitant increase in Bacteroidota (*p* < 0.05), resulting in a significant reduction in the B/B ratio (*p* < 0.05) (**Figures 6A, B**). Following HAMSIAA administration, the B/B ratio of the fecal microbiota was maintained at a level comparable to that of the NC group (*p* < 0.05), suggesting that HAMSIAA effectively attenuated CTX-driven microbial imbalance and preserved community homeostasis compared with the CTX group (**Figures 6C–E**).

Beyond the core community shifts, at the phylum level, Pseudomonadota was significantly enriched in the CTX group compared with the NC group, whereas this enrichment was attenuated in the HAMSIAA group (**Figure 6A**). Consistently, at the class level, Gammaproteobacteria (the dominant class within Pseudomonadota) was significantly expanded in the CTX group. However, this increase was attenuated following HAMSIAA intervention (**Figure 6B**).

### 3.7 HAMSIAA attenuated CTX-induced metabolic disorders in mice

**Figure 7:**
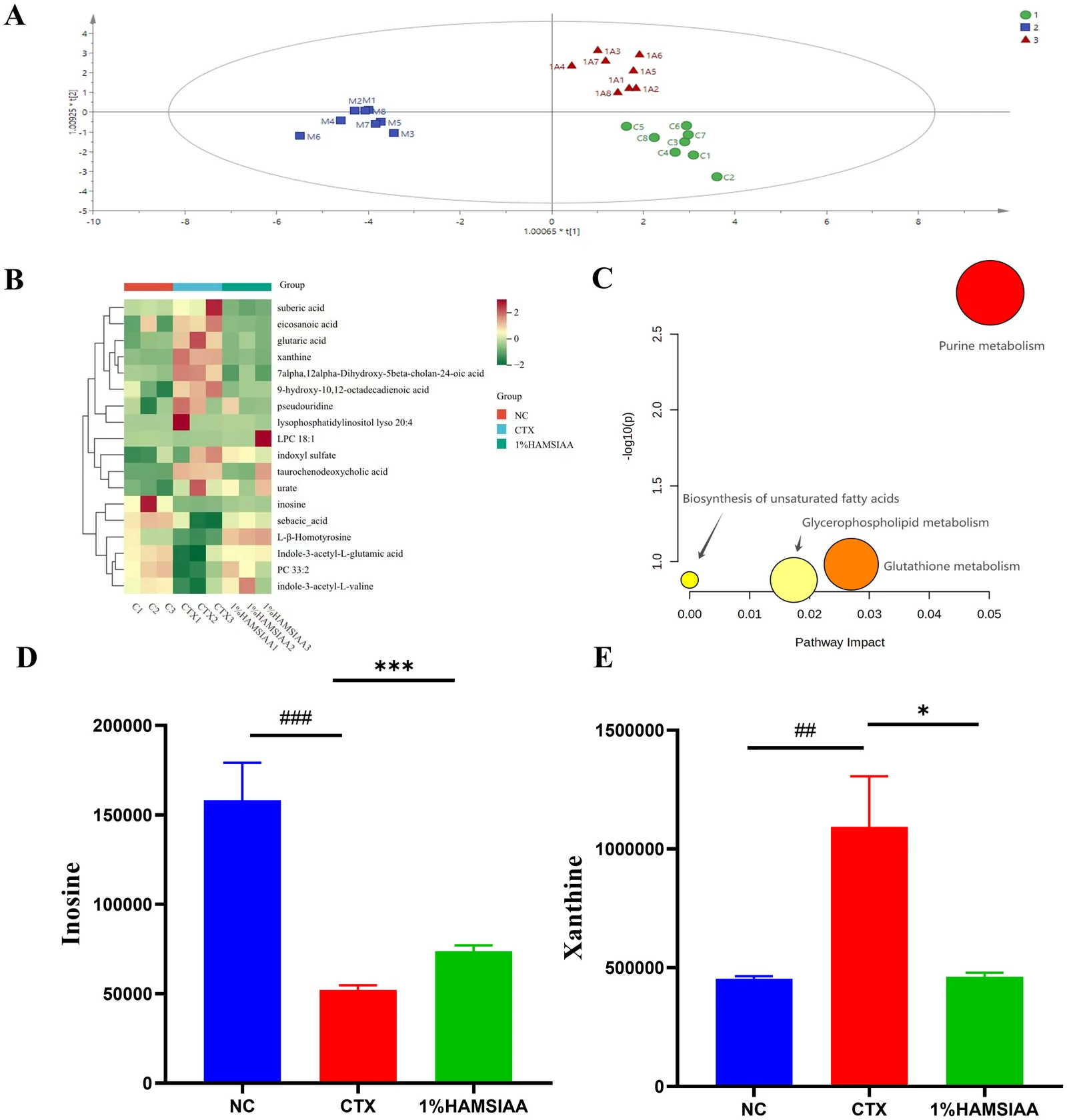
Effects of HAMSIAA on the serum metabolome in CTX-induced immunocompromised mice. **(A)** OPLS-DA score plot. **(B)** Heatmap of differential metabolites among the NC, CTX, and 1% HAMSIAA groups (n=3). **(C)** KEGG pathway enrichment analysis. **(D)** Serum inosine levels (n=8). **(E)** Serum xanthine levels (n=8). Data are presented as mean ± SEM. ^##^*p* < 0.05, ^##^*p* < 0.01, ^###^*p* < 0.001 versus NC group; *p < 0.05, **p < 0.01, ***p < 0.001 versus CTX group.

Untargeted metabolomics was performed to investigate the effects of HAMSIAA on the serum metabolic profiles of CTX-induced immunosuppressed mice. Untargeted metabolomics analysis was performed on serum samples from the NC, CTX, and 1% HAMSIAA groups (n = 8 per group). OPLS-DA revealed clear separation among the NC, CTX, and HAMSIAA groups, with good intra-group clustering, indicating strong explanatory and predictive power of the model (**Figure 7A**). Subsequently, differentially abundant metabolites among groups were analyzed. Compared with the NC group, 10 metabolites showed increased abundance and 10 showed decreased abundance in the CTX group. Compared with the CTX group, 8 metabolites showed increased abundance and 11 showed decreased abundance in the HAMSIAA group (**Figure 7B**). Kyoto Encyclopedia of Genes and Genomes (KEGG) pathway enrichment analysis was performed to elucidate the biological functions of these differentially abundant metabolites. This analysis identified four enriched pathways: purine metabolism, glutathione metabolism, biosynthesis of unsaturated fatty acids, and glycerophospholipid metabolism, among which purine metabolism was the most significantly enriched pathway (**Figure 7C**). Differential metabolite analysis further demonstrated that HAMSIAA intervention significantly regulated key purine metabolites, as evidenced by increased serum inosine levels and decreased xanthine levels (**Figures 7D, E**), suggesting that purine metabolic changes may be involved in the immunomodulatory effects of HAMSIAA, although causality remains to be further validated.

### 3.8 HAMSIAA modulated key enzyme expression in the purine salvage pathway

**Figure 8:**
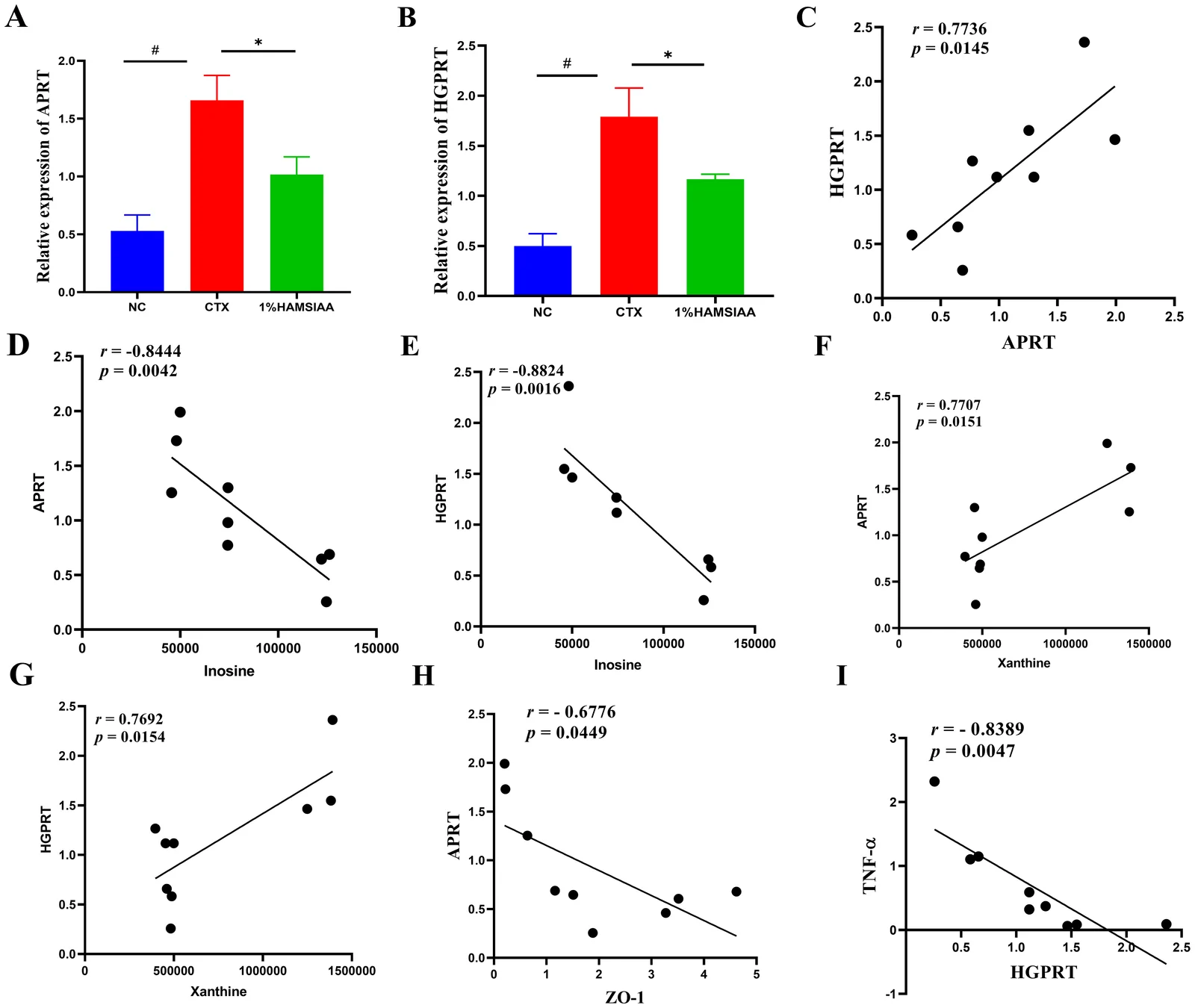
Effect of HAMSIAA on mRNA expression and correlation of purine metabolism-related genes in thymus tissues of CTX-induced immunocompromised mice. **(A)** *APRT* mRNA expression. **(B)** *HGPRT* mRNA expression. **(C)** Correlation between *APRT* and *HGPRT* expression. **(D)** Correlation between serum inosine and thymic *APRT* expression. **(E)** Correlation between serum inosine and thymic *HGPRT* expression. **(F)** Correlation between serum xanthine and thymic *APRT* expression. **(G)** Correlation between serum xanthine and thymic *HGPRT* expression. **(H)** Correlation between *ZO-1* and *APRT* expression. **(I)** Correlation between *TNF-α* and *HGPRT* expression. All data are based on the NC, CTX, and 1% HAMSIAA groups (n = 3 per group). For panels A and B, data are presented as mean ± SEM. ^#^*p* < 0.05, ^##^*p* < 0.01, ^###^*p* < 0.001 versus NC group; *p < 0.05, **p < 0.01, ***p < 0.001 versus CTX group. For panels **(C–I)**, correlation coefficients were calculated using Pearson correlation analysis.

Compared with the NC group, the mRNA expression levels of *APRT* and *HGPRT* in the thymus of CTX-induced immunosuppressed mice were significantly increased (*p* < 0.05). However, intervention with 1% HAMSIAA significantly downregulated their expression compared with the CTX group (*p* < 0.05) (**Figures 8A, B**). Correlation analyses were performed to evaluate the relationships among key genes in the thymus of the NC, CTX, and 1% HAMSIAA groups. A significant positive correlation was observed between *HGPRT* and *APRT* expression (r = 0.7736, p = 0.0145) (**Figure 8C**).

To evaluate the association between systemic purine metabolic changes and transcriptional responses in immune organs, we further analyzed the correlations of serum inosine and xanthine with thymic *APRT* and *HGPRT* expression. In the NC, CTX, and 1% HAMSIAA groups, inosine showed negative correlations with *APRT* and *HGPRT*, whereas xanthine showed positive correlations with *APRT* and *HGPRT* (**Figures 8D–G**). Notably, inosine was significantly negatively correlated with *HGPRT* (r = −0.8824, p = 0.0016), while xanthine was significantly positively correlated with *HGPRT* (r = 0.7692, p = 0.0154). It should be noted that these analyses are cross-compartmental and therefore exploratory; the observed correlations suggest systemic metabolic-immune crosstalk rather than direct local causality.

Furthermore, ileal *ZO-1* expression was significantly negatively correlated with thymic *APRT* expression in the NC, CTX, and 1% HAMSIAA groups (r = −0.6776, p = 0.0449) (**Figure 8H**); ileal *TNF-α* expression was significantly negatively correlated with thymic *HGPRT* expression in the 1% HAMSIAA group (r = −0.8389, p = 0.0047) (**Figure 8I**).

## 4 Discussion

The immune system serves as a critical defense barrier for maintaining systemic homeostasis by recognizing and eliminating invading pathogens while preserving self-immune tolerance (15, 16). In recent years, the prevalence of immunodeficiency has increased, driven by factors including dietary changes, environmental pollution, lifestyle modifications, psychological stress, and medication use. Immunosuppression compromises the body’s defensive capacity, increasing susceptibility to pathogenic infections and impairing apoptotic cell clearance and tumor surveillance (17), which may further lead to respiratory or gastrointestinal infections, fatigue, and an elevated risk of tumorigenesis (18). Therefore, the development of safe and effective interventions to counteract immune dysfunction remains of considerable importance for maintaining host health.

CTX-induced immunosuppression is typically characterized by weight loss, immune organ atrophy, reduced immunoglobulin levels, and dysregulated immune cell distribution. Previous studies have reported that serum IgA, IgG, and IgM levels are significantly decreased in CTX-induced immunosuppressed mice, whereas treatment with *Dictyophora echinovolvata* spore polysaccharides significantly elevated these immunoglobulins, suggesting enhanced humoral immunity (19). Our findings were consistent with this pattern: HAMSIAA attenuated CTX-induced immune organ atrophy and elevated serum IgA, IgG, and IgM levels. We next examined splenic T-lymphocyte subsets, which are critical for adaptive immune surveillance (20). CD8^+^ T cells are major mediators of cellular immunity, directly eliminating abnormal cells and secreting cytokines to enhance host defense (21). The percentage of CD4^+^ T cells and the CD4^+^/CD8^+^ ratio are widely used as indicators of immune function, as they reflect the balance between helper and cytotoxic T-cell populations (22, 23). A decreased CD4^+^/CD8^+^ ratio is closely associated with immunosuppression and an elevated risk of infection (23, 24). HAMSIAA significantly increased the percentages of CD4^+^ and CD8^+^ T cells as well as the CD4^+^/CD8^+^ ratio. These results are consistent with previous observations that immunomodulatory substances effectively attenuate T-lymphocyte subset perturbations in CTX-treated mice (25). Similarly, Zhang et al. reported that American ginseng increased splenic CD4^+^CD8^-^ T cells and attenuated the reduction in the CD4^+^/CD8^+^ ratio in CTX-treated mice (26). Thus, modulation of T-cell subsets appears to be a shared mechanism among natural immunomodulators. B cells produce antibodies and maintain humoral immune homeostasis (27). HAMSIAA did not significantly affect B-cell proportions, suggesting that the observed increase in immunoglobulin levels likely reflects enhanced B-cell function rather than an expansion of B-cell populations.

The intestinal mucosal barrier is crucial component for maintaining immune homeostasis, and its integrity depends on tight junction proteins, including Occludin and ZO-1 (28). Previous studies have demonstrated that CTX downregulates ileal Occludin and ZO-1 expression (29, 30). Our results were consistent with these reports: CTX-induced immunosuppression reduced their expression, whereas HAMSIAA attenuated this effect. The histological score further supported these findings, which showed that HAMSIAA significantly ameliorated CTX-induced tissue injury. Cytokines coordinate innate and adaptive immunity (31). Notably, HAMSIAA modulated the mucosal immune balance by regulating the balance between pro-inflammatory (IL-1β, TNF-α) and anti-inflammatory (IL-10) cytokines. This pattern aligns with previous reports showing that immunostimulatory agents simultaneously elevate both arms of the cytokine response in CTX-induced immunosuppression models (32, 33).

To explore the underlying mechanism, we examined AhR pathway activation in the ileum. Our data showed that HAMSIAA significantly upregulated ileal *CYP1A1* mRNA expression and elevated IL-22 protein levels, confirming activation of the IAA-AhR axis. These results link the elevated fecal IAA induced by HAMSIAA to AhR activation in the ileum, supporting the involvement of this pathway in the immunomodulatory effects of HAMSIAA on intestinal mucosal immunity.

**Figure 9:**
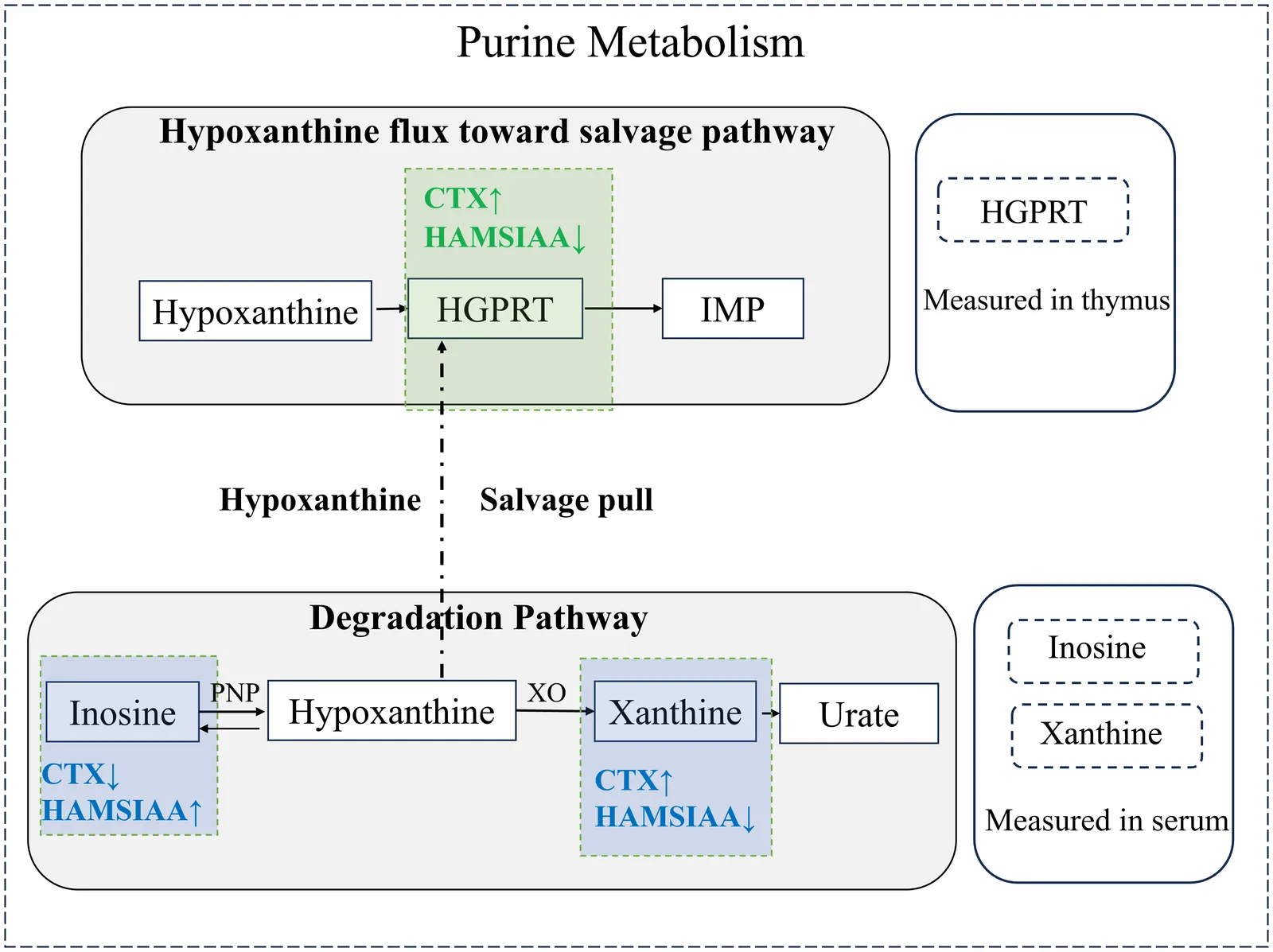
Schematic model illustrating the putative interplay between thymic HGPRT expression and purine metabolite abundance identified via untargeted metabolomics upon HAMSIAA intervention. This schematic proposes a correlative metabolic hypothesis rather than definitive causal regulation. The salvage pathway (upper panel) displays HGPRT expression quantified in thymic tissues. The degradation pathway (lower panel) presents signature purine metabolites (inosine and xanthine) enriched through untargeted metabolomic profiling. Purine nucleoside phosphorylase (PNP) catalyzes the bidirectional interconversion of inosine and hypoxanthine, whereas xanthine oxidase (XO) drives the oxidation of hypoxanthine to xanthine. Arrows denote molecular alterations triggered by CTX (vs. normal control group) and HAMSIAA (vs. CTX model group): CTX upregulated HGPRT protein and xanthine abundance while decreasing inosine abundance, and HAMSIAA rescued these abnormal metabolic phenotypes. The dashed arrow depicts the presumed metabolic flux of hypoxanthine shunted into the salvage pathway via HGPRT. XO enzymatic activity was not directly quantified in the present study. Dashed boxes distinguish two independent sample matrices: thymic tissue for target protein quantification and metabolomic specimens for purine metabolite profiling.

We further examined the regulatory effects of HAMSIAA on intestinal microbial communities. CTX treatment markedly disrupted microbial ecology, as evidenced by an aberrant increase in Chao1 richness, enrichment of Pseudomonadota (a biomarker of dysbiosis), a reduced B/B ratio, and expansion of Bacteroidota and Muribaculaceae. The elevated Chao1 reflects a “diversity paradox’’ reported in certain chemotherapy settings (34), wherein CTX preferentially suppresses dominant commensals and releases ecological niches for opportunistic taxa, rather than a broadly beneficial diversification. HAMSIAA significantly attenuated these dysbiotic features, including the expansion of Pseudomonadota and CTX-induced increase in Chao1, while also elevating the B/B ratio, a corrective shift away from the dysbiotic state. Although interpretation of the B/B ratio is context-dependent (35), its elevation by HAMSIAA in this model is consistent with restoration of microbial balance rather than a pathological change. We also note that while these microbial shifts correlate with improved immune parameters, our data do not establish that the gut microbiota directly mediates the immunoprotective effects of HAMSIAA, and future studies using antibiotic depletion, fecal microbiota transplantation (FMT), or gnotobiotic models will be required to establish causality.To elucidate the molecular basis of HAMSIAA-mediated immunoprotection, untargeted metabolomics was performed, revealing that prophylactic HAMSIAA administration substantially modulated purine metabolism. Given the established link between purine salvage and immune homeostasis, we focused on HGPRT as a potential regulatory node. Previous studies have associated elevated HGPRT expression with immunosuppression, suggesting that its upregulation under pathological conditions may represent an adaptive response to evade immune surveillance (36). Consistent with this, CTX-treated mice exhibited marked HGPRT overexpression in the thymus, whereas HAMSIAA significantly downregulated thymic HGPRT. These local transcriptional changes were accompanied by systemic metabolic shifts: HAMSIAA increased serum inosine and decreased circulating xanthine. Pearson correlation analysis further suggested a metabolic-immune crosstalk, showing that thymic HGPRT expression was negatively correlated with serum inosine and positively correlated with serum xanthine. Mechanistically, HGPRT upregulation promotes hypoxanthine consumption via the salvage pathway, shifting the equilibrium of the purine nucleoside phosphorylase (PNP)-catalyzed reaction between inosine and hypoxanthine toward inosine degradation. This is consistent with the reduced serum inosine in CTX-treated mice. Conversely, HAMSIAA-mediated downregulation of HGPRT would reduce hypoxanthine consumption, allowing inosine accumulation. This interpretation is grounded in established purine biochemistry, with hypoxanthine serving as a pivotal branch node between salvage and degradation. The changes in xanthine may involve distinct mechanisms, potentially related to xanthine oxidase (XO) activity, although XO expression was not measured in this study. Collectively, these findings suggest that HAMSIAA attenuates CTX-triggered immunosuppression in association with modulation of the purine salvage pathway centered on HGPRT. However, these correlative findings are exploratory, and future studies with tissue-matched metabolomics and direct enzyme activity measurements will be required to validate these associations. A schematic model integrating these findings is presented in **Figure 9**.

Correlation analysis further revealed associations between purine metabolism-related genes and both immune parameters and barrier function. Notably, APRT expression was negatively correlated with ZO-1 across the analyzed groups, while HGPRT expression was also negatively correlated with TNF-α. Although these observations hint at potential links between thymic purine metabolism and intestinal barrier or immune function, they are exploratory and do not establish causality.

Several limitations should be noted. This study used a single model with male mice only, and the microbiota findings are correlational rather than causal. Multi-omics and correlation analyses were limited to the 1% HAMSIAA group with small sample sizes, and some measurements involved cross-compartment comparisons. Functional barrier assays and protein-level cytokine data were not included. Despite these limitations, our findings provide a solid basis for developing HAMSIAA as a prophylactic immunomodulatory dietary ingredient.

## 5 Conclusion

Taken together, our results suggest that HAMSIAA effectively attenuated CTX-triggered immunosuppression in mice, in association with multi-layered regulatory pathways: protection of immune organ function, preservation of intestinal barrier integrity, maintenance of immune cell balance, remodeling of gut microbial composition, and reprogramming of purine metabolism. These observations lay a robust experimental foundation for developing HAMSIAA as a novel functional dietary component for the prophylactic mitigation of chemotherapy-related immune impairment.

## Data Availability

The raw sequencing data presented in this study have been deposited in the NCBI Sequence Read Archive (SRA) database under BioProject accession number PRJNA1519798. The BioSample accession numbers for the individual samples are SAMN62743602–SAMN62743619.
