# Supplementary material for: HAMSIAA protects cyclophosphamide induced immunosuppression by modulating gut homeostasis and purine metabolism
Source: Front Immunol. 2026 Sep 3;17:1925823. doi: 10.3389/fimmu.2026.1925823 (PMC13581576; doi:10.3389/fimmu.2026.1925823)
Supplement: Supplementary file 1 [file Table1.docx]

Table 1. Primer sequeces for qRT-PCR

| Gene Name | Primer Type(F/R) | Primer Sequence (5'-3') |
| --- | --- | --- |
| GAPDH | F | CATCACTGCCACCCAGAAGACTG |
| GAPDH | R | ATGCCAGTGAGCTTCCCGTTCAG |
| IL-1β | F | TGGACCTTCCAGGATGAGGACA |
| IL-1β | R | GTTCATCTCGGAGCCTGTAGTG |
| TNF-α | F | GGTGCCTATGTCTCAGCCTCTT |
| TNF-α | R | GCCATAGAACTGATGAGAGGGAG |
| APRT | F | GTCATTGTGGATGACCTCCTGG |
| APRT | R | TGGTATAGGTCCTAGCCTCC |
| HGPRT | F | CCTGGCGTCGTGATTAGTGAT |
| HGPRT | R | AGACGTTCAGTCCTGTCCATAA |
| ZO-1 | F | GTTGGGTACGGTGCCTGAAAGA |
| ZO-1 | R | GCTGACAGGGTAGGACAGACGAT |
| Occludin | F | TGGGCAAGCATCATACCCAGAG |
| Occludin | R | CTGCCTGAAGTCATCCACACTC |
| CYP1A1 | F | GACCCTTACAAGTATTTGGTCGT |
| CYP1A1 | R | GGTATCCAGAGCCAGTAACCT |
